# A Case Study of Pharmaceutical Pricing in China: Setting the Price for Off-Patent Originators

**DOI:** 10.1007/s40258-014-0150-5

**Published:** 2015-06-20

**Authors:** Shanlian Hu, Yabing Zhang, Jiangjiang He, Lixia Du, Mingfei Xu, Chunyan Xie, Ying Peng, Linan Wang

**Affiliations:** School of Public Health, Fudan University, 138 Yi Xue Yuan Rd, Shanghai, 200032 People’s Republic of China; Shanghai Health Development Research Center, Shanghai, People’s Republic of China; Shanghai Institute of Technology, Shanghai, People’s Republic of China; Division of Health Legislation and Regulation, Shanghai Health and Family Planning Commission, Shanghai, People’s Republic of China

## Abstract

This article aims to define a value-based approach to pricing and reimbursement for off-patent originators using a multiple criteria decision analysis (MCDA) approach centered on a systematic analysis of current pricing and reimbursement policies in China. A drug price policy review was combined with a quantitative analysis of China’s drug purchasing database. Policy preferences were identified through a MCDA performed by interviewing well-known academic experts and industry stakeholders. The study findings indicate that the current Chinese price policy includes cost-based pricing and the establishment of maximum retail prices and premiums for off-patent originators, whereas reference pricing may be adopted in the future. The literature review revealed significant differences in the dissolution profiles between originators and generics; therefore, dissolution profiles need to be improved. Market data analysis showed that the overall price ratio of generics and off-patent originators was around 0.54–0.59 in 2002–2011, with a 40 % price difference, on average. Ten differentiating value attributes were identified and MCDA was applied to test the impact of three pricing policy scenarios. With the condition of implementing quality consistency regulations and controls, a reduction in the price gap between high-quality off-patent products (including originator and generics) seemed to be the preferred policy. Patents of many drugs will expire within the next 10 years; thus, pricing will be an issue of importance for off-patent originators and generic alternatives.

## Key Points for Decision Makers

**Table:** 

Patents of many drugs will expire within the next 10 years; therefore, favorable pricing and purchasing strategies have to be developed to optimize access to affordable drugs while simultaneously incentivizing drug financing and a sustainable supply.
This study encompasses a review of current pricing and reimbursement policies for off-patent originator and generic drugs in China.
A literature review revealed current gaps in the quality assurance and control of generics in China.
A market data analysis revealed a consistent average price difference of approximately 40 % between generics and originators.

## Background

### The Drug Pricing System in China

In China, pharmaceuticals are differentiated into three classes, namely patented drugs, off-patent drugs (originators), and generics. Between 2002 and 2004, a total of 14,392 generics were approved by the China Food and Drug Administration (CFDA) [1]. As a result of China not following the zero tax policy for imported drugs, set by the World Trade Organization, a 5 % import tax as well as a 17 % value-added tax are included in the price of all imported drugs.

An analysis of the Chinese pharmaceutical market from 2010 to 2020 by the IMS Institute for Healthcare Informatics projected that traditional Chinese medicines will constitute 11 % of the total market value and that the value share of patented drugs will increase from 6 to 11 % [Dr. Dehui Han, IMS Health, personal communication (2012)]. The share of off-patent originators is expected to continue at approximately 20 %, with the remaining 60 % of the market being dominated by generics.

In China, drug pricing is managed by both central and provincial governments. However, the government can only control the price of drugs reimbursed by the basic medical insurance schemes, as well as of some narcotic drugs [2]. Initial patented drug prices can be freely set by the manufacturers since drugs can only be listed for medical insurance reimbursement after 2 years of market availability, following which the government controls the maximum retail price. Nevertheless, market competition between enterprises is allowed. China’s drug prices are set and administrated by the Bureau of Pricing of the National Development and Reform Commission (NDRC). The transaction price can be reduced through bulk procurement and tender bidding systems, and each province establishes a drug tender bidding center [3]. Currently, many provinces have a ‘two-envelope selective tender system’ in place [4], in which the tenderers place the drug quality information, as well as the price, into two separate sealed envelopes. The bidding center then uses the first envelope to select the three highest quality drugs as candidates, and the process is completed by opening of the second envelope to compare their prices. Thus, of the three selected manufacturers, the one offering the lowest price will win the tender. However, bidding procedures are not standardized, and the consideration of drug prices alone, without prior quality prioritization, can occur. Furthermore, if the bidding price is below the real production cost, manufacturers are unable to produce and supply the drugs, leading to drug shortages. Finally, the bid price is not related to either purchasing or volume [5]. Thus, bidding procedures in China are in dire need of improvement. Recently, Chinese government plans to formulate a drug reference pricing system have been suggested; such a system would impact the price of both domestic generics and off-patent drugs.

### Evaluation of Quality Consistency

Differences between off-patent originators and generics in China can arise from variations in quality management, medicine quality, and therapeutic equivalence. The Chinese 12th Five-Year Plan for Drug Safety (2011–2015) noted some quality gaps within generic products compared with international standards, which can influence both clinical efficacy and safety [6]. For example, the Shanghai CFDA detected such gaps for both imported and domestic generics, where the rate of substandard quality was <0.1 and 3 %, respectively [7]. Thus, the CFDA promulgated the Amended Regulation on the Administration of Drug Registration in 2007, through which generics licensed before 2007 need to be evaluated against originators for quality and consistency. A total of 205 essential medicines, involving approximately 30,000 certificates of approval, will be certified within the 12th Five-Year Plan (2010–2015) [8, 9].

### Literature Review of Generics Quality in China

Reviews of comparative studies evaluating the quality and consistency of seven drugs were retrieved, including cefaclor [10], fluconazole (Diflucan^®^) [11], irbesartan [12], telmisartan (Micardis^®^) [13], loratadine [14], simvastatin [15], and aspirin [16]. These studies were published in Chinese journals from 2001 to 2012, and involved 63 domestic pharmaceutical companies. Significant differences in the dissolution profiles of originators and their generic versions were observed (Table 1). These findings highlight the urgency of improving the quality of generics produced by some domestic manufacturers. We hope that the present evaluation of quality consistency will help improve the control of substandard generics produced by some domestic pharmaceutical companies. Furthermore, we suggest that the Chinese government should consider an incentive policy for manufacturers whose generics have passed quality consistency evaluations. Such incentives could include granting of a premium price, listing in medical insurance reimbursement schemes, and awarding of a favorable procurement review policy. In the short-term, most domestic generics manufacturers will face challenges concerning quality and consistency. In contrast, off-patent originator manufacturers will be given the opportunity to expand their market share until domestic manufacturers have caught up. Although these incentives will not reduce pharmaceutical expenditure in the short term, long-term benefits include generics quality improvement and a strengthened competitiveness. Thus, in the long-term, off-patent originator manufacturers may be forced to reduce their premium prices in order to remain among the preferred suppliers [17].

**Table 1:** Comparative study on dissolution profiles of some drugs

Comparator (manufacturer)	No. of generic pha rmaceutical companies in China	Experiment buffer conditions	Conclusions	References
Cefaclor capsules (Eli Lilly)	11	Three buffer solutions: pH 1.2, 4.0, and 6.8, and water	Significant differences among the quality of cefaclor capsules from different manufacturers; thus, the quality of generic drugs needs to be improved	Gu and Hu [10]
Loratadine tablets (Schering Plough)	33	Four types of dissolution media with varying pH values	Overall, 105 dissolution profiles were obtained, with great in vitro differences observed; only the results of four enterprises were consistent	Liu et al. [14]
Fluconazole capsules (Pfizer)	2	Hydrochloric acid solution (0.1 mol/L), water, and phosphoric acid buffer (pH 6.8)	In 0.1 mol/L hydrochloric acid solution and phosphoric acid buffer (pH 6.8), the dissolution profile of a given generic is different from the original. However, in water, both generics are significantly different from that of the original product. Their formulation and process of preparation should be improved	Yan et al. [11]
Telmisartan tablets (Boehringer Ingelheim)	5	Three dissolution media, 0.1 mol/L hydrochloric acid solution, phosphate buffer (pH 7.5)	Products from two pharmaceutical companies were significantly different compared with the original drug	Yang et al. [13]
Irbesartan tablets (Sanofi-aventis)	3	Four different media: pH 1.2 hydrochloric acid, pH 4.0 acetate buffer, pH 6.8 phosphate buffer, and water	There were significant differences between the dissolution profiles of brand name and generic drugs	Jia et al. [12]
Simvastatin capsules (Merck)	9	Four types of media: 0.5 % SDS-water, 0.5 % SDS-artificial gastric juice pH 1.2, 0.5 % SDS-acetate buffer pH 4.0, and 0.5 % SDS-phosphate buffer pH 7.0	There were significant differences in quality among the simvastatin capsules from different manufacturers; thus, the quality of domestic simvastatin capsules needs to be improved	Li et al. [15]
Aspirin (Bayer)	5	Acidic medium: 0.1 mol/L hydrochloric acid and pH 6.8 phosphate buffer solution	The dissolution rates of all five domestically-produced enteric-coated aspirin tablets were remarkably rapid	Lv et al. [16]

*SDS* sodium dodecyl sulfate

In China, full implementation of quality consistency assessments will be a long-term process. It has been suggested that quality assessments should include the three levels of equivalence, i.e., pharmaceutic, therapeutic, and bioequivalence [18]. Pharmaceutical equivalence refers to the chemical consistency evaluation of the active pharmaceutical ingredients. Bioequivalence refers to pharmacokinetics and pharmacodynamics, as well as in vitro testing, in order to ensure interchangeability of generics versus the originator or reference product. Therapeutic equivalence testing includes clinical studies comparing efficacy and safety in clinical trials and clinical practice. Currently, in China, only a dissolution profile is required to test the quality consistency between generics and patented (originator) drugs. Nevertheless, even if the dissolution curves are consistent, therapeutic and bioequivalence may differ [19].

### Potential Changes in Off-Patent Originator Pricing Policy

In July 2010, the NDRC promulgated measures for the regulation and administration of drug prices with the goal of implementing a consistent pricing policy and to encourage investment in further research and development for innovative drugs [20]. These drugs would have to fulfill criteria for a differential pricing policy, such as patented drugs, off-patent originators, confidential formulations of traditional Chinese medicines, and first generic drugs entering the market approved by the US FDA and the European Medicines Agency, or authorized by the Chinese FDA, including high-quality generics that passed the quality consistency evaluation.

Drug pricing in China is gradually being transferred from cost-based to clinical value-based pricing, along with a shift from the highest retail price to medical insurance payment reference pricing [21]. These measures were implemented in some pilot provinces in 2014. The reference price is set by the Bureau of Pricing of the NDRC and Ministry of Human Resources and Social Security and defines the maximum level of payment or reimbursement for each drug class. The difference between the actual end price and the reference price will have to be paid by the patient. Thus, competition between pharmaceutical companies supplying high-quality generics and off-patent originators is encouraged with the objective of arriving at rational drug prices. Other measures that are being considered for drug pricing include the introduction of pharmacoeconomic evaluation, an international reference pricing mechanism, and price negotiations [22].

## The Pricing Trend Relating to Off-Patent Originators in China

To compare the use of off-patent originators and generics in China’s domestic market, market-share data were analyzed for patented drugs, off-patent originators, and generics. These data were collected from the Shanghai Purchasing Drug Information System between 2002 and 2011, and included information from 120 hospitals (32 tertiary hospitals, 73 secondary hospitals, and 15 community hospitals) throughout eastern China and the Yangzi river area provinces [17]. The monthly database included 588,300 records, which were merged into a full year. The price trend and ratio were also analyzed. The results indicated an increasing trend of generics prescribing, coupled with approximately 300 generic forms of off-patent originators entering the Chinese domestic market each year. Furthermore, half of the generic dosage forms were tablets and capsules, and one-third were injections.

The overall price ratio of generics to their off-patent originators was found to be in the range of 0.54–0.59 (95 % confidence interval 0.49–0.62) from 2002 through 2011, indicating that the average relative price difference was stable, at approximately 40 % (Table 2). However, there were also some drugs with larger differences, and some generic versions were even more expensive than the off-patent originators [17].

**Table 2:** Price ratio between off-patent and generic drugs in China (2002–2011)

Year	No. of drugs	Mean^a^	95 % CI	SD	Median	Range
2002	144	0.54	0.49–0.58	0.26	0.54	0.02–1.15
2003	167	0.55	0.51–0.59	0.26	0.57	0.02–1.21
2004	199	0.55	0.52–0.59	0.24	0.58	0.02–1.14
2005	226	0.58	0.55–0.62	0.26	0.61	0.02–1.56
2006	262	0.58	0.55–0.62	0.28	0.61	0.02–2.07
2007	273	0.56	0.53–0.60	0.26	0.59	0.02–1.39
2008	286	0.56	0.53–0.59	0.26	0.59	0.02–1.58
2009	296	0.58	0.55–0.61	0.27	0.62	0.02–1.64
2010	303	0.59	0.56–0.62	0.27	0.64	0.03–1.63
2011	306	0.58	0.55–0.61	0.26	0.60	0.02–1.55

*CI* confidence interval, *SD* standard deviation

^a^Values correspond to the ratio of generic to off-patent originator drug prices

**Fig. 1:**
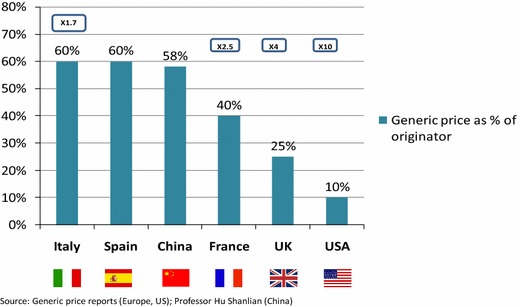
Comparison of price between originator and generics in six countries. × denotes the factor of the price difference between originator and generics, e.g. ×10 indicates that, in the US, the generics price corresponds to 10 % of the originator price. Source: Abbott Co

The price relationship from generic to originator drugs (generics price as a percentage of originator price) was comparable to those in Italy and Spain (both 60 %) but less pronounced than in France (40 %), UK (25 %), and the US (10 %). Overall, the international price ratios between generics and off-patent originators varied two to tenfold (Fig. 1). However, without a quality and efficacy comparison, these price ratios can only be used as a reference for future price adjustments.

Currently, off-patent originator prices are coming under increasing scrutiny. With the use of tender and bulk-purchasing processes in some Chinese provinces, off-patent originators can no longer obtain or maintain their premium prices. Given the improvement in the quality of generics certified by the CFDA with current Good Manufacturing Practice (GMP) or by the Japanese GMP controlled by the Japanese Ministry of Health, Labour, and Welfare, some generics are now considered interchangeable with off-patent originators. Alternative to using tenders to determine the price of off-patent originator drugs, negotiation could facilitate the agreement of both parties to a final price.

## Can Multiple Criteria Decision Analysis be Used to Set Off-Patent Originator Prices?

Multiple criteria decision analysis (MCDA) is a tool that can be applied to complex decisions involving a choice among alternatives, and establishes preferences between options by applying an explicit set of objectives [23]. Current MCDA approaches compare the alternatives with regard to their impact on the different criteria, and require an exercise of judgment. Formal MCDA techniques usually include the step of criteria weighting to provide for the varying degrees of importance of each criterion [24].

In November 2013, an international group of health economists and health policy experts in the pharmaceutical field^1^ came together to develop a method of using MCDA to evaluate policies for off-patent originators and generic products. The product attributes that may determine the value of off-patent products in China were identified through a stakeholder consultation meeting and a key opinion leader teleconference.

The key attributes considered to impact the achievement of healthcare objectives, and thus potentially useful for the pricing of off-patent originators and generics in China, were (i) pharmaceutical equivalence related to the quality consistency evaluation of active ingredients (currently required for the quality of Chinese generics); (ii) bioequivalence (currently not required for Chinese generics); (iii) a new 2010 Chinese version of GMP certification for pharmaceutical manufacturing enterprises; (iv) clinical evidence of efficacy and effectiveness (important evidence for value-based pricing); (v) drug safety; (vi) patient adherence to therapy; (vii) differences in excipients (e.g. salts, esters, ethers, isomers, mixtures of isomers, complexes, or derivatives), coatings, and production processes and technology, all of which may have an influence on physicochemical and clinical stability and product shelf-life; (viii) order of entry in the market; (ix) supply reliability in the Chinese market; and (x) manufacturer investment in China (e.g. joint-venture manufacturing partnerships).

Thus, three possible pricing policies would be considered by the Chinese government. First, if the quality of Chinese generic products has been proven, using the quality consistency evaluation, to be significantly similar to the original drugs, the pricing gap between the off-patent originators and generics will be reduced (Policy 1). Second, value-based pricing could be chosen on the grounds of MCDA (Policy 2). Third, the NDRC and the Ministry of Human Resources and Social Security will operate a reference pricing system which defines the reimbursement level; patients would pay the price difference between the retail price at the hospital or pharmacy and the defined reference price (Policy 3).

For the MCDA survey in China, 11 well-known academic experts and 7 senior pharmaceutical staff were interviewed and asked to fill in an MCDA questionnaire. They first determined the relative importance (weight 1–10) of each of the ten attributes and then scored the impact of each of the three policy conditions on each attribute (weight 1–5). The MCDA tool was adapted from an international health economics expert initiative sponsored by Abbott Products Operations AG (Switzerland). The score of MCDA estimates (weight 0–1) was calculated based on each alternative policy. Finally, the results gave an indication of which pricing policy would best achieve the objectives of the Chinese stakeholders.

The number of weights for each attribute is shown in Table 3. The results were very similar for both groups. There were five attributes with scores higher than the average (0.1), and the ranked order of the first three was clinical efficacy and effectiveness, drug safety, and bioequivalence. Order of market entry, supply reliability, and investment were not considered to be important for off-patent originator pricing. Regarding the different pricing-policy scenarios, the academics preferred Policy 1 (total score of 0.72), followed by Policy 2 (0.70) and Policy 3 (0.62). Pharmaceutical staff also preferred Policy 1 (0.72), followed by Policy 3 (0.69) and Policy 2 (0.67) (Table 3). These results indicate that both groups are most likely to consider pharmaceutical equivalence first. The information collected from the MCDA questionnaire survey and a focus group discussion (20 persons) held in Shanghai showed that academic experts were not in favor of a reference pricing policy due to concerns that this new pricing policy would increase a patient’s economic burden. The pharmaceutical staff group thought a reference pricing approach would be good for driving market competition. Thus, although value-based pricing, coupled with MCDA, may be used for pricing, both groups had concerns regarding its feasibility.

**Table 3:** The weights and scores of each attribute in different interview groups

Attributes	No. of weights		Academia group scores			Pharma group scores		
	Academia group (*n* = 11)	Pharma group (*n* = 7)	Policy 1^a^	Policy 2^b^	Policy 3^c^	Policy 1^a^	Policy 2^b^	Policy 3^c^
1. Pharmaceutical equivalence	0.11	0.11	0.91	0.78	0.60	0.89	0.69	0.83
2. Bioequivalence	0.12	0.11	0.87	0.80	0.69	0.91	0.69	0.71
3. Accreditation of pharmaceutical enterprises by the new version of the 2010 Chinese GMP	0.10	0.10	0.58	0.53	0.55	0.83	0.63	0.69
4. Clinical efficacy and effectiveness	0.12	0.14	0.75	0.85	0.64	0.77	0.89	0.83
5. Drug safety	0.12	0.12	0.82	0.85	0.78	0.74	0.86	0.77
6. Patient adherence to therapy	0.10	0.09	0.76	0.82	0.62	0.83	0.69	0.69
7. Different excipients, production process and technology, shelf-life	0.11	0.11	0.62	0.69	0.71	0.51	0.69	0.69
8. Order of entry in the market	0.09	0.09	0.64	0.55	0.44	0.49	0.37	0.60
9. Supply reliability	0.08	0.08	0.53	0.53	0.58	0.49	0.54	0.46
10. Manufacturer investment	0.06	0.05	0.51	0.36	0.36	0.54	0.31	0.34
MCDA estimates			0.72	0.70	0.62	0.72	0.67	0.69

^a^Policy 1 refers to the possible reduction in price difference between off-patent originators and Chinese generic products if the quality of both remains consistent

^b^Policy 2 refers to the choice of value-based pricing

^c^Policy 3 refers to the choice of a reference pricing system for reimbursement

## Policy Alternatives

### The Challenge of Off-Patent Originator Pricing

Pharmaceutical equivalence requirements ensure the quality of generics in China appears realistic. In general, the quality of off-patent originators was found to be better than the quality of generics. Nevertheless, even if the pharmaceutical equivalence of the generic formulation has been proven through quality consistency evaluation, there is no data to confirm the consistency with regard to therapeutic and bioequivalence. In addition, superior production processes, technologies, ingredients (such as excipients and coatings), and other factors may be expected to contribute not only to a drug’s shelf-life but also to its overall quality. Thus, during the bidding system and bulk-purchasing process, premium pricing-level maintenance or inclusion in a higher quality medicine category should be considered for off-patent originators.

### Improvement of the Pricing Policy for Off-Patent Originators

Several countries worldwide are continuing drug pricing policy reviews and reforms in order to improve population equity and access to drugs whilst remaining within the limits of the available budgets. The Chinese government is also aiming to expand coverage of essential medicines while improving the overall quality of drug choices [25]. A gradual change from cost pricing to value-based pricing may provide a scientific and rational drug pricing policy for off-patent originators. The value of a pharmaceutical drug is driven by multiple criteria, such as clinical efficacy, safety, and patient compliance, along with the improvement of patient quality of life and ethical and social benefits. Therefore, these factors should be considered when prioritizing and rewarding drugs in drug formularies, and when formulating new price classification systems for all pharmaceutical drugs and biomedicines in China.

The government should consider reducing drug price control measures and increasing incentives for market competition as a good pricing mechanism. Thus, the market could be opened to drugs that are not included in the medical insurance drug reimbursement list, provided that there is a market need for such drugs.

### Creating a Balanced Market for Patented Drugs, Off-Patent Originators, and Generics

At present, the goal in China’s pharmaceutical market is to achieve a balanced access across patented drugs, off-patent originators, and generics. Generic drugs dominate the market as copies of off-patent originators. Based on the IMS Institute for Healthcare Informatics pharmaceutical market analysis and projection for the years 2010–2020, the compound annual growth rate in the value of patented drugs, off-patent originators, and generics is 24, 19, and 15 %, respectively.

There is still a need to establish a defined basket of reference countries comparable to China’s economic status. The quality consistency evaluation of generic drugs should continue to be a priority and include those with premium prices. Manufacturers of patented drugs should be encouraged to enter into price negotiation with third-party payers. Finally, the future pricing administration should emphasize the relationship between price and quality, as well as the price ratio between off-patent originators and generics.

## Conclusions

This study encompasses a review of current pricing and reimbursement policies for off-patent originators and generic drugs as applied in China. Clinical value-based and reference pricing seem to be the future trends for drug price setting. A literature review revealed current gaps in the quality assurance of domestic generics in China; specifically, there are significant differences between the dissolution profiles of off-patent originators and domestic generics. Therefore, we suggest that the quality of some generic products needs to be improved.

A Chinese market data analysis performed between 2002 and 2011 illustrated a consistent average price difference of approximately 40 % between off-patent originators and generics; this price ratio should be used as a reference for future price adjustment. Within the coming 10 years, the patent protection of many patented drugs will expire and will lead to the production of off-patent originators. Although these off-patent drugs are of good quality, further challenges and opportunities, such as the value differentiation between off-patent originators and generics, will be faced. Along with improvements in the quality-consistency assessments of Chinese domestic generics, the price gap between off-patent originators and generics will also be diminished. Thus, optimal pricing and purchasing strategies have to be developed while simultaneously incentivizing sustainable quality and supply; this will ensure a balanced market access between patent drugs, off-patent originators, and generics. Furthermore, value and price setting among the three drug categories will become an important and interdependent issue.

The MCDA method was successfully used herein to prioritize possible future pricing and reimbursement strategies for off-patent drugs, as tested by Chinese academic and industry experts. The results revealed that both academics and industry stakeholders are most likely to consider pharmaceutical equivalence first. The study limitations include the small sample size and the lack of direct involvement of political policymakers within the survey. However, policymakers should consider the use of MCDA for policy decision making; additionally, value-based pricing policymaking requires greater awareness and recognition in the near future.

## Footnotes

1 Participants: S.L. Hu (China), M. Bosi-Ferraz (Brazil), Z. Kaló (Hungary), E.L. Toverud (Norway), D. Brixner (USA), N. Maniadakis (Greece), R. Alfonso (Columbia and USA), I.V. Koshkarova (Russia), S. Gonzalez-McQuire (Switzerland), J. Shen (Switzerland), and A.P. Holtorf (Switzerland).
